# Learning from our mistakes

**DOI:** 10.1080/24740527.2017.1378568

**Published:** 2017-10-23

**Authors:** Manfred Harth

**Affiliations:** Department of Rheumatology, University of Western Ontario, London, Ontario, Canada

**Keywords:** Fibromyalgia, Linden, patient contribution

## Abstract

The article by M.-A. Fitzcharles et al. appearing in this issue represents an attempt to elicit suggestions from a group of patients with fibromyalgia (FM) and a group of health professionals on the leading uncertainties in the treatment of FM. The sample of respondents in both these groups is not adequately representative, the methodology used is unduly complex, and the responses obtained do not represent new or useful information.

## RÉSUMÉ

L’article de M.-A. Fitzcharles et al. publié dans ce numéro décrit une tentative de recueillir les suggestions d’un groupe de patients souffrant de fibromyalgie (FM) et d’un groupe de professionnels de la santé en ce qui concerne les principales incertitudes dans le traitement de la FM. L’échantillon de répondants dans les deux groupes n’est pas suffisamment représentatif, la méthodologie utilisée est indûment complexe et les réponses obtenues ne contiennent pas d’information nouvelle ou utile.

## Introduction

The James Lind Alliance (JLA) is an initiative launched in the United Kingdom in 2004, designed to involve patients with clinicians in agreeing on priorities for research on the effects of treatment.^1^ Its advocates have pointed out that the uncertainties and concerns of patients and clinicians in such research have usually been ignored in the past.^2^ The current issue of this journal includes a paper by Fitzcharles et al., who used the JLA methodology to explore the indications for potential research in the treatment of fibromyalgia.^3^ Fibromyalgia (FM) is a common condition characterized by widespread pain, fatigue, nonrefreshing sleep, and cognitive difficulties. It disables many and is often poorly understood by both patients and health care professionals.^4^ To date its treatment has been only modestly successful.^4,5^

The article by Fitzcharles et al.^3^ illustrates the difficulties in reaching a useful and clear agreement between patients and health care professionals for a treatment research agenda. The work, the time, and the number of people involved in this project were considerable, but the validity and originality of the results presented appear questionable. The main problems are listed below.

### Missing data and uneven representation in the responder group

There were 550 responders to the survey but in only 73% of cases could they be identified as either “patient” or “clinician.” Thirteen responders were identified as “organization members,” another 14 were labeled as “caregivers,” and in 122 responders there was “no identification” (Table 2 in Fitzcharles et al.^3^). It appears, nevertheless, that even responses from the latter three groups were considered in subsequent analyses. In the patient group, there was overrepresentation from Quebec and underrepresentation from Ontario. There were 109 clinician responders, of whom 45% were rheumatologists. Only 12% of the clinicians were family physicians, and there were only seven psychologists and only four psychiatrist responders.

### The diagnosis of FM

The diagnosis of FM was self-reported in this study. In a recent National Health Interview Survey in the United States, 73.5% of those self-reporting this diagnosis did not satisfy the National Health Interview Survey criteria for FM.^6^

### Subjective elements in the process of uncertainty formulation

The JLA process involved submitting to the respondents open-ended questions, modeled on previous studies but with a focus on FM. The authors do not provide the wording used in those questions. The responses received were then reviewed, and answers irrelevant to the topic of treatment uncertainties were discarded. Then questions on treatment answered by previous research were also discarded. This left a set of 25 questions (not further detailed in the paper), which were then discussed in workshops and further reduced to ten questions to be considered for research. Though the process involved in these endeavors was laborious, there were clearly strong subjective elements involved in these various steps.

### The finalists

Ten priorities for research were eventually identified (Table 4 in Fitzcharles et al.^3^). Each of these is briefly labeled as a source of uncertainty and several questions are cited for clarification under the heading of “sample narrative.” The differentiation between the priorities is not always clear. Thus, research uncertainties 4 and 9 both relate to education and information. Research uncertainty 6 also seems to be similar to 4 and 9 above in that it focuses on education and information by video or social media. The authors discuss at some length the uncertainty with respect to “cannabinoids” and opioids. There should be no uncertainty about one opioid—namely, tramadol—which has been used with moderate success in FM.^5^ There have been no good double-blind long-term randomized trials of opioids in FM. In view of current concerns with respect to the harmful effects of such medications when used for the treatment of chronic non-cancer pain, such a trial for FM is unlikely in the foreseeable future.^7^ Cannabinoids such as nabilone have been used in FM with questionable results.^5^ Many physicians and patients have been waiting for appropriate good quality trials with marijuana in FM, but neither the companies involved in the distribution of medical marijuana nor third parties such as nonprofit research organizations have indicated any interest so far.

The authors show considerable interest in what they term “the first theme”—namely, that of “individualized treatment approaches”—which two of them (Fitzcharles and Häuser with D. J. Clauw as a coauthor) had already discussed in a recent publication.^8^ They acknowledged in that paper that such a treat-to-target approach would require considerable advances in our knowledge before such an approach could be considered.^8^

The authors refer to a “second theme” of uncertainty; namely, examination of self-management strategies such as lifestyle modifications, educational techniques, and methods to improve health literacy. Education is important in the treatment of FM, but it is not equivalent to lifestyle modifications that would include modalities such as aerobic and strengthening exercise, cognitive behavioral therapy (CBT), or CBT combined with exercise.^4,5^ Though CBT and exercise can be eventually self-managed, they will require expert introductory instructions for most patients. Interestingly, exercises such as yoga and tai chi, for which there is some evidence of benefit in the literature^9^ but that require further study, are not included in the list of uncertainties.^3^

## Final remarks

The JLA is s right in promoting patient participation in setting research priorities and planning. This will of necessity be laborious. It may not always yield useful results, and I believe this to be the case here. The authors have acknowledged some methodological deficiencies. Others to be considered are the use of open questions and the interpretation of the responses by workshop participants, thus adding to the subjectivity of the process. A preset quota of 25 uncertainties that is then reduced to a final ten may be too high. Complexity is not necessarily a virtue. Future investigators on patient contributions to research should read this paper carefully to avoid making the same mistakes.
